# Safety of SARS-CoV-2 vaccination during pregnancy- obstetric outcomes from a large cohort study

**DOI:** 10.1186/s12884-022-04505-5

**Published:** 2022-02-28

**Authors:** Aharon Dick, Joshua I Rosenbloom, Einat Gutman-Ido, Naama Lessans, Adva Cahen-Peretz, Henry H Chill

**Affiliations:** 1 Department of Obstetrics and Gynecology, Hadassah Medical Organization, Faculty of Medicine, Hebrew University of Jerusalem, Jerusalem, 91120 Israel; 2 Division of Urogynecology, University of Chicago Pritzker School of Medicine, NorthShore University HealthSystem, Skokie, IL USA

**Keywords:** SARS-CoV-2, Outcomes, Pregnancy, Preterm birth, Safety, Small for gestational age, Trimester, Vaccination

## Abstract

**Background:**

COVID-19 during pregnancy is associated with adverse outcomes for mother and fetus. SARS-CoV-2 vaccination has significantly reduced the risk of symptomatic disease. Several small studies have reported the safety of SARS-CoV-2 vaccination during pregnancy, with no adverse effect on obstetric outcomes.

**Objective:**

To examine the association between SARS-CoV-2 vaccination during pregnancy and maternal and neonatal outcomes in a large cohort study. Furthermore, to evaluate if timing of vaccination during pregnancy is related to adverse outcomes.

**Methods:**

A retrospective cohort study of women who delivered between December 2020 and July 2021 at a large tertiary medical center. Excluded were women with multiple pregnancy, vaccination prior to pregnancy, COVID-19 infection during or before pregnancy, or unknown timing of vaccination. Primary outcomes were the incidence of preterm labor and of small for gestational age. Secondary outcomes were other maternal and neonatal complications. A secondary analysis investigating the association between time of vaccination and outcomes was also performed. Multivariable logistic regression models were used to adjust for potential confounders.

**Results:**

There were 5618 women who met the inclusion criteria: 2,305 (41%) women were vaccinated and 3,313 (59%) were unvaccinated. There were no differences between vaccinated and non-vaccinated patients with respect to primary outcomes. The rate of preterm birth was 5.5% in the vaccinated group compared to 6.2% in the unvaccinated group (*p* = 0.31). Likewise, the rates of small for gestational age were comparable between the two groups (6.2% vs. 7.0% respectively, *p* = 0.2).

In a secondary analysis focusing on time of vaccination and its relationship with outcomes, patients vaccinated in the second trimester (*n* = 964) and in the third trimester (*n* = 1329) were independently compared to their unvaccinated counterparts. Women who were vaccinated in the second trimester were more likely to have a preterm birth (8.1% vs. 6.2%, *p* < 0.001). This association persisted after adjusting for potential confounders (adjusted odds ratio 1.49, 95%CI 1.11, 2.01).

**Conclusions:**

SARS-CoV-2 vaccine appears to be safe during pregnancy with no increase in incidence of preterm labor and small for gestational age compared to unvaccinated women. However, in women vaccinated during the second trimester there may be an increase in the rate of preterm birth.

## Background

The COVID-19 pandemic, with more than 4 million deaths worldwide, has been a major source of concern over the past 2 years [1]. Pregnant individuals are a population at increased risk for severe disease with implications on both mother and fetus [2]. Several studies have demonstrated increased rates of preterm birth and low birthweight among women infected with SARS-CoV-2 during pregnancy [3–5]. These adverse outcomes have been observed even in healthy women with no co-morbidities [6].

In late 2020 the SARS-CoV-2 vaccine was introduced, first by Pfizer BioNTech (BNT162b2) and later by Moderna (mRNA-1273). Vaccination was shown to result in a greater than 85% reduction in the risk of symptomatic disease and its transmission [7]. Although the vaccine was not tested in pregnant women, the Centers for Disease Control and Prevention (CDC) [8] and the American College of Obstetricians and Gynecologists (ACOG) [9] recommend its use among pregnant women.

Several studies have demonstrated the safety of SARS-CoV-2vaccine in pregnancy, showcasing a protective effect from symptomatic disease, with minimal adverse neonatal outcomes [10–13]. Moreover, some studies have suggested that maternal vaccination may induce offspring immunity against SARS-CoV-2 [14, 15]. However, little is known regarding the possible effect of timing of vaccination during pregnancy, since the majority of women were vaccinated in the third trimester.

Therefore, the aim of our study was to evaluate maternal and neonatal outcomes in women who received the SARS-CoV-2 vaccine during pregnancy in comparison to women who remained unvaccinated. We also examined how time of vaccination during pregnancy may be associated with these outcomes.

## Methods

 This is a retrospective cohort study conducted Hadassah-Hebrew University Center in Jerusalem, a large tertiary-care center with two campuses. Women with singleton deliveries from December 2020 until July 2021 were included. Exclusion criteria included multiple pregnancy, vaccination prior to pregnancy, COVID-19 infection during or before pregnancy, or unknown timing of vaccination.

Our center maintains a comprehensive delivery database. Available clinical and demographic data included maternal age, obstetric history, maternal comorbidities, smoking, body mass index, and COVID-19 status, defined as vaccinated, infected, both vaccinated and infected, or neither vaccinated nor infected. During the time of the study the vaccine used in Israel was either the Pfizer BioNTech (BNT162b2) or Moderna vaccines. The co-primary outcomes were preterm birth, defined as birth before 37 weeks gestation, and small for gestational age, defined as birthweight below the tenth percentile for gestational age and sex using local birthweight standards [16]. Gestational age was determined by the last menstrual period and the earliest ultrasound performed in pregnancy. Gestational diabetes and hypertensive disorders of pregnancy were defined according to the criteria of the American College of Obstetrics and Gynecology [17].

Secondary outcomes included gestational age at delivery, birthweight, stillbirth, mode of delivery (cesarean or vaginal), postpartum hemorrhage (defined as estimated blood loss > 500 mL), and neonatal outcomes including 5-minute Apgar score, and umbilical artery pH and base excess when available. Our hospital does not have a universal cord gas collection policy. Adverse neonatal outcomes included 5-minute Apgar score < 7 or umbilical cord arterial pH < 7.1.

To examine if there was a difference in outcomes associated with timing of vaccination receipt, we conducted a secondary analysis, comparing outcomes for patients who received the first dose of the vaccine in the second trimester versus the third trimester. In this analysis, the reference case was defined as unvaccinated patients.

### Statistical analysis

Descriptive statistics including medians and interquartile ranges were used to describe the cohort. Baseline characteristics between vaccinated and unvaccinated patients were compared using the Mann Whitney U test or Kruskall-Wallis test for continuous variables and the chi-square or Fisher’s exact test for categorical variables. Potential confounders for an association between vaccination and the outcomes were identified using directed acyclic graphs. Then, multivariable logistic regression adjusting for potential confounders including maternal age, body mass index (BMI), nulliparity, and smoking was performed. The association between vaccination and hypertensive disorders of pregnancy and gestational diabetes was also evaluated, although data on the timing of these diagnoses relative to the date of vaccination were not available.

## Results

**Fig. 1:**
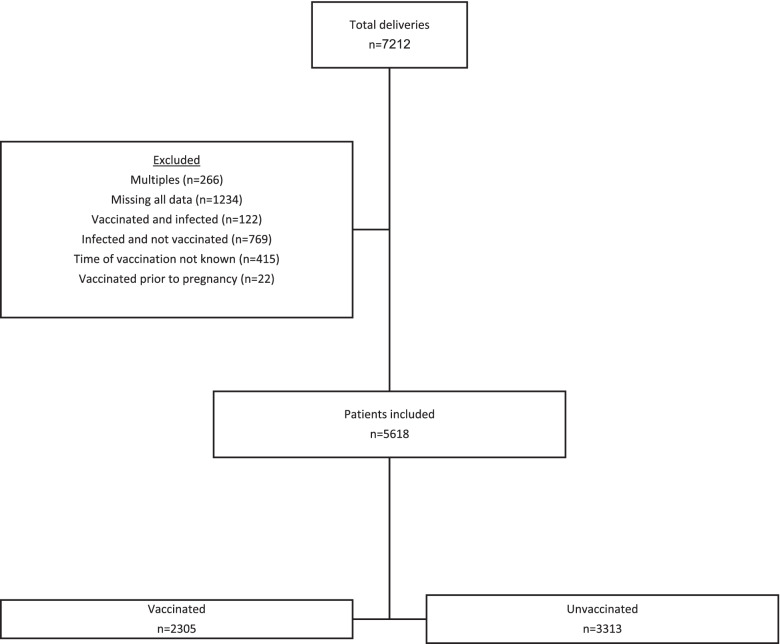
Study Flow Diagram

Overall there were 7,212 deliveries during the study period. After exclusions, there were 5,618 patients available for analysis, of whom 2,305 (41%) were vaccinated and 3,313 (59%) were unvaccinated (Fig. 1). Baseline characteristics of the patients are seen in Table 1. Vaccinated women had a slightly higher median body mass index, but otherwise there were no significant differences between the groups.

**Table 1:** Baseline Characteristics and Outcomes of Vaccinated vs. Unvaccinated Patients

	Vaccinated *n* = 2305	Unvaccinated *n* = 3313	p
Maternal age	30 (26, 34)	30 (26, 34)	0.009
Body mass index (kg/m^2^)	26.4 (23.4, 30.1)	25.6 (22.2, 29.4)	< 0.001
Parity	1 (0,3)	2 (0,3)	0.27
Nulliparous	611 (26.5)	838 (25.3)	0.31
Smoking	79 (3.4)	88 (2.7)	0.09
Primary Outcomes			
Preterm birth	127 (5.5)	204 (6.2)	0.31
Small for gestational age	142 (6.2)	233 (7.0)	0.20
Secondary outcomes			
Gestational age at delivery, weeks	39.7 (38.5, 40.4)	39.7 (38.7, 40.4)	0.33
Cesarean delivery	358 (15.5)	529 (16.0)	0.66
Postpartum hemorrhage (> 500 mL)	79 (3.4)	104 (3.1)	0.55
Intrauterine fetal demise	20 (0.87)	33 (1.0)	0.62
Birthweight, g	3280 (2980, 3590)	3260 (2955, 3575)	0.083
Apgar 5 min	10 (10,10)	10 (10,10)	0.63
5 min Apgar < 7	42 (1.8)	63 (1.9)	0.83
Umbilical arterial pH	7.28 (7.21, 7.34)	7.28 (7.22, 7.34)	0.32
Umbilical pH < 7.1	89 (7.4)	122 (6.8)	0.57
Umbilical arterial base excess	-4.1 (-6.0, -2.7)	-3.9 (-5.7, -2.5)	0.007
Hypertensive disorder of pregnancy	25 (1.1)	44 (1.3)	0.42
Gestational diabetes	222 (9.6)	275 (8.3)	0.08

Data are median (interquartile range) or n (%)

**Fig. 2:**
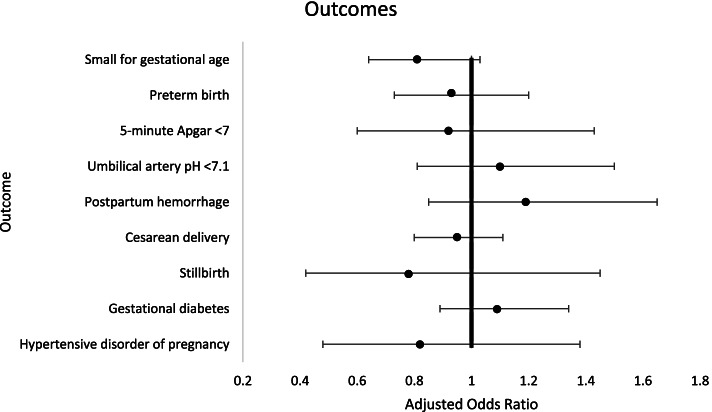
Adjusted Odds Ratios and 95% Confidence Intervals for Vaccinated vs. Unvaccinated Parturients

There were no differences between vaccinated and non-vaccinated patients in the primary outcomes (Table 1) (rate of preterm birth in the vaccinated group 5.5% vs. 6.2% in the unvaccinated group, *p* = 0.31, rate of small for gestational age 6.2% vs. 7.0%, *p* = 0.20). Among the secondary outcomes there were also no significant differences except for a slightly lower umbilical cord base excess in vaccinated patients (-4.1 vs. -3.9, *p* = 0.007). The results of the multivariable analysis are shown in Fig. 2. After adjusting for potential cofounders such as nulliparity, maternal age, body mass index and maternal smoking, women vaccinated were not at increased risk for small for gestational age, preterm birth, postpartum hemorrhage or cesarean delivery. Similarly, there were no significant differences between the two groups in neonatal outcomes such as 5 min Apgar score, low umbilical PH or stillbirth rates.

Of the vaccinated patients, 12 (0.5%) were vaccinated in the first trimester and were excluded from the secondary analysis. Of the remaining vaccinated women, 964 (41.8%) were vaccinated in the second trimester and 1,329 (57.7%) were vaccinated in the third. There were no significant differences in baseline characteristics for patients vaccinated in the second vs. the third trimester (Table 2). However, patients vaccinated in the second trimester were more likely to have a preterm birth, and this association persisted after adjusting for potential confounders (adjusted odds ratio 1.49, 95%CI 1.11, 2.01), compared to unvaccinated patients, Tables 3 and 4. The median gestational age at preterm birth in the patients vaccinated in the second trimester was 35 weeks (IQR 34.1. 36.6), while for unvaccinated patients the median gestational age at preterm birth was 35.1 weeks (IQR 32.1, 36.3), *p* = 0.024. The median delay from first vaccination to preterm delivery in patients who were vaccinated in the second trimester and delivered preterm was 14.3 weeks (IQR 11.3, 18.4). There were no patients who were vaccinated in the second trimester and delivered within two weeks of vaccine receipt.

**Table 2:** Baseline Characteristics by Trimester of Vaccination

	Second Trimester *n* = 964	Third Trimester *n* = 1329	p
Maternal age	30 (26, 34)	30 (26, 35)	0.32
Body mass index (kg/m^2^)	26.3 (23.4, 30.1)	26.5 (23.4, 30.1)	0.63
Parity	1 (0,3)	1 (0,3)	0.44
Nulliparous	256 (26.6)	354 (26.6)	0.97
Smoking	31 (3.2)	48 (3.6)	0.61

Data are median (interquartile range) or n (%)

**Table 3:** Outcomes by Trimester of Vaccination

Outcome	Second Trimester *n* = 964	Third Trimester *n* = 1329	Unvaccinated *n* = 3313	p
Primary Outcomes				
Preterm birth	78 (8.1)	40 (3.0)	204 (6.2)	< 0.001
Small for gestational age	58 (6.0)	82 (6.2)	233 (7.0)	0.39
Secondary outcomes				
Gestational age at delivery, weeks	39.4 (38.4, 40.3)	39.9 (38.6, 40.6)	39.7 (38.7, 40.4)	0.0012
Cesarean delivery	159 (16.5)	198 (14.9)	529 (16.0)	0.54
Postpartum hemorrhage (> 500 mL)	35 (3.6)	43 (3.2)	104 (3.1)	0.75
Intrauterine fetal demise	10 (1.0)	4 (0.3)	33 (1.0)	0.05
Birthweight, g	3250 (2930, 3560)	3315 (3000, 3618)	3260 (2955, 3575)	0.0012
Apgar 5 min	10 (10, 10)	10 (10, 10)	10 (10,10)	0.29
5 min Apgar < 7	16 (1.7)	20 (1.5)	63 (1.9)	0.63
Umbilical arterial pH	7.28 (7.21, 7.35)	7.28 (7.21, 7.34)	7.28 (7.22, 7.34)	0.37
Umbilical pH < 7.1	46 (9.1)	42 (6.0)	122 (6.8)	0.10
Umbilical arterial base excess	-4.3 (-6.2, -2.9)	-4.0 (-5.9, -2.5)	-3.9 (-5.7, -2.5)	0.0054
Hypertensive disorder of pregnancy	8 (0.8)	16 (1.2)	44 (1.3)	0.46
Gestational diabetes	104 (10.8)	116 (8.7)	275 (8.3)	0.06

Data are median (interquartile range) or n (%)

**Table 4:** Adjusted Odds Ratios and 95% Confidence Intervals by Vaccination Trimester

Outcome	Second Trimester	Third Trimester	Unvaccinated
Primary Outcomes			
Preterm birth	1.49 (1.11, 2.01)	0.49 (0.34, 0.71)	*referent*
Small for gestational age	0.73 (0.52, 1.03)	0.85 (0.64, 1.13)	*referent*
Secondary Outcomes			
Cesarean delivery	1.05 (0.85, 1.30)	0.88 (0.72, 1.07)	*referent*
Postpartum hemorrhage (> 500 mL)	1.11 (0.71, 1.74)	1.22 (0.83, 1.78)	*referent*
Intrauterine fetal demise	1.12 (0.52, 2.38)	0.25 (0.08, 0.82)	*referent*
5 min Apgar < 7			*referent*
Umbilical pH < 7.1	1.46 (0.99, 2.14)	0.88 (0.60, 1.30)	*referent*
Hypertensive disorder of pregnancy	0.71 (0.33, 1.54)	0.83 (0.45, 1.55)	*referent*
Gestational diabetes	1.21 (0.93, 1.58)	0.99 (0.77, 1.27)	*referent*

Odds ratios adjusted for nulliparity, maternal age, body mass index, and maternal smoking

## Discussion

In this study we describe obstetric outcomes following SARS-CoV-2 vaccination during pregnancy. Apart for a slightly increased umbilical arterial base excess level, women in the vaccinated group had comparable maternal and fetal outcomes compared to unvaccinated women. However, following analysis of outcomes according to time of vaccination, women who were vaccinated during the second trimester had increased rate of preterm birth compared to unvaccinated women.

Our findings correlate partially with previous studies. Wainstock et al. reported on a cohort of 4399 pregnant women out of which 913 (20.6%) received either one or two vaccines during pregnancy. No differences were found between groups in pregnancy, delivery and fetal outcomes [10]. In another study, data on 1328 pregnant women of whom 140 received at least one dose of the SARS-CoV-2 vaccine were presented showing no adverse maternal or fetal outcomes in the vaccinated group [11]. In a third study, 712 women who received two doses of the SARS-CoV-2 vaccine were compared to 1063 unvaccinated women. SARS-CoV-2 vaccination was not associated with maternal composite adverse outcomes and was shown to reduce risk of neonatal adverse composite outcomes [18]. Similar to these findings, in our primary analysis we did not detect any adverse outcomes in vaccinated women. This included both maternal and neonatal outcomes, pointing towards vaccine safety.

To date the question of how timing of vaccination may affect pregnancy outcomes has yet to be addressed. Understandably, most previous studies have not had sufficient sample size to investigate this variable. Furthermore, in most of these studies SARS-CoV-2 vaccination was administered during the third trimester, with few women receiving the vaccine during the first or second trimesters. We found women who were vaccinated during the second trimester had an increased risk of preterm birth compared to their unvaccinated counterparts (8.1% vs. 6.2%, *p* < 0.001). Notably, there were no preterm births within two weeks after vaccine receipt in the second trimester, and the majority of the preterm births were in the late preterm period, suggesting that unmeasured confounding may have contributed to the results.

Strengths of this study include the investigation of the association between time of vaccination and pregnancy outcomes. Furthermore, this is the largest comparative study to date focusing on maternal and fetal outcomes following vaccination.

Apart from its retrospective design, this study has several limitations. We were unable to report on outcomes of vaccination during first trimester of pregnancy due to small sample size. Allocation to trimester was according to time of first vaccine. Hence, there were women in the second trimester group who received their second dose during the third trimester leading to possible overlap between groups. We excluded women who were infected with COVID-19, since their outcomes have been widely reported elsewhere. However, an unvaccinated patient is at increased risk of infection with its consequent potential obstetric outcomes. Additionally, data regarding adverse events following vaccination was unavailable. Finally, we had limited data on risk factors for preterm birth.

## Conclusions

In this cohort study, SARS-CoV-2 vaccine appears to be safe during pregnancy with no increase in incidence of preterm labor and small for gestational age compared to unvaccinated women. However, in women vaccinated during the second trimester there may be an increase in the rate of preterm birth. Future studies focusing on outcomes according to time of vaccination may enable a better understanding and more accurate patient counseling on vaccination risks and benefits during pregnancy.

## Data Availability

The datasets used and/or analyzed during the current study are available from the corresponding author on reasonable request.

## Abbreviations

COVID-19: coronavirus disease of 2019
SARS-CoV-2: Severe acute respiratory syndrome coronavirus 2
